# Spatiotemporal Small Non-coding RNAs Expressed in the Germline as an Early Biomarker of Testicular Toxicity and Transgenerational Effects Caused by Prenatal Exposure to Nanosized Particles

**DOI:** 10.3389/ftox.2021.691070

**Published:** 2021-06-29

**Authors:** Satoshi Yokota, Ken Takeda, Shigeru Oshio

**Affiliations:** ^1^Division of Cellular and Molecular Toxicology, Center for Biological Safety and Research, National Institute of Health Sciences, Kawasaki, Japan; ^2^Division of Toxicology and Health Science, Faculty of Pharmaceutical Sciences, Sanyo-Onoda City University, Yamaguchi, Japan; ^3^Department of Hygiene Chemistry, School of Pharmaceutical Sciences, Ohu University, Koriyama, Japan

**Keywords:** testicular toxicity, transgenerational effects, epigenetics, nanomaterial, inhalation, developmental origins of health and disease (DOHaD)

## Abstract

In recent years, an apparent decline in human sperm quality has been observed worldwide. One in every 5.5 couples suffers from infertility, with male reproductive problems contributing to nearly 40% of all infertility cases. Although the reasons for the increasing number of infertility cases are largely unknown, both genetic and environmental factors can be contributing factors. In particular, exposure to chemical substances during mammalian male germ cell development has been linked to an increased risk of infertility in later life owing to defective sperm production, reproductive tract obstruction, inflammation, and sexual disorders. Prenatal exposure to nanomaterials (NMs) is no exception. In animal experiments, maternal exposure to NMs has been reported to affect the reproductive health of male offspring. Male germ cells require multiple epigenetic reprogramming events during their lifespan to acquire reproductive capacity. Given that spermatozoa deliver the paternal genome to oocytes upon fertilization, we hypothesized that maternal exposure to NMs negatively affects male germ cells by altering epigenetic regulation, which may in turn affect embryo development. Small non-coding RNAs (including microRNAs, PIWI-interacting RNAs, tRNA-derived small RNAs, and rRNA-derived small RNAs), which are differentially expressed in mammalian male germ cells in a spatiotemporal manner, could play important regulatory roles in spermatogenesis and embryogenesis. Thus, the evaluation of RNAs responsible for sperm fertility is of great interest in reproductive toxicology and medicine. However, whether the effect of maternal exposure to NMs on spermatogenesis in the offspring (intergenerational effects) really triggers multigenerational effects remains unclear, and infertility biomarkers for evaluating paternal inheritance have not been identified to date. In this review, existing lines of evidence on the effects of prenatal exposure to NMs on male reproduction are summarized. A working hypothesis of the transgenerational effects of sperm-derived epigenomic changes in the F1 generation is presented, in that such maternal exposure could affect early embryonic development followed by deficits in neurodevelopment and male reproduction in the F2 generation.

## Introduction

Infertility is a major health issue worldwide. Today, infertility in Japan affects one in every 5.5 couples, a trend that has been observed in other populations as well. Of these, male factors contribute to infertility in >40% of cases (De Kretser and Baker, 1999; Hotaling and Carrell, 2014; Lotti and Maggi, 2018). Causes of male infertility can be classified as (1) defective sperm production, (2) reproductive tract obstruction, (3) inflammation, and (4) sexual disorders (De Kretser, 1997). Approximately half of the cases of male infertility result from defective sperm production (i.e., abnormal sperm motility and morphology, complete arrest of spermatogenesis, low sperm count, etc.) (De Kretser and Baker, 1999). Although the etiology of most cases remains unknown, there is increasing evidence that apart from genetic defects, environmental factors are also a significant cause of male infertility (Sharpe, 2010).

A number of chemical substances are present in the environment, and humans are routinely exposed to them. In particular, a fetus is so vulnerable to environmental factors that prenatal and neonatal exposure can result in more adverse effects later in life than adult exposure, even if the exposure occurs at low levels. This may also be true for endocrine-disrupting chemicals such as vinclozolin (Anway et al., 2006; Guerrero-Bosagna et al., 2012), 2,3,7,8-tetrachlorodibenzo[p]dioxin (Manikkam et al., 2012), and plastic-derived endocrine disruptors in animal experiments (Manikkam et al., 2013). However, unlike the studies on the developmental toxicity of endocrine-disrupting chemicals, there have been very few animal studies on delayed toxicological effects of maternal exposure to particulate matter (PM), including nanomaterials (NMs), in later stages of life. From the perspective of animal studies, this review covers the various windows of susceptibility during mammalian male germ cell development (F1) and shows that prenatal NM exposure can influence spermatogenesis in the offspring. Additionally, exposure to NMs during the fetal period has been shown to cause intergenerational (F1) and transgenerational (F2) effects on daily sperm production in mice (Kyjovska et al., 2013). Thus, we discuss the possibility that the impact of maternal exposure to NMs on spermatogenesis in the F1 generation can trigger paternally induced effects on subsequent generations.

## Development of the Testis

**Figure 1:**
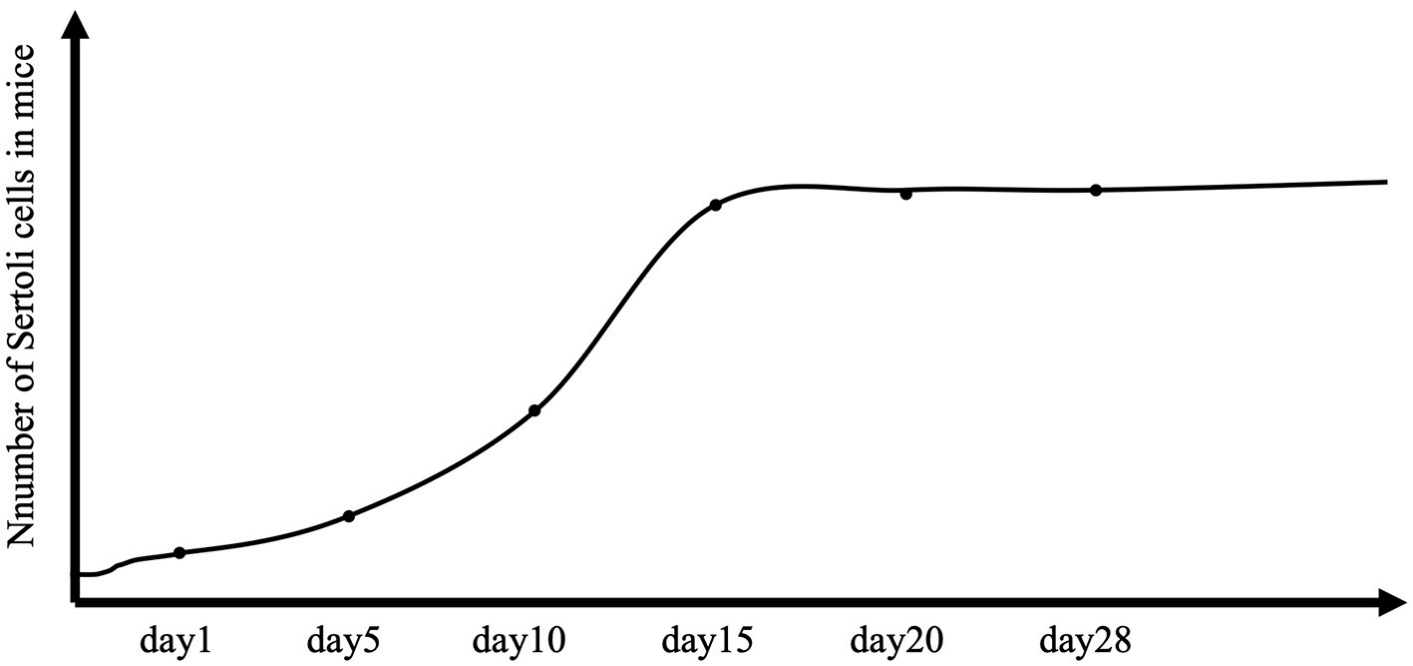
Total number of Sertoli cells per testis in controls. The number of Sertoli cells per testis is considered stable throughout the life of the animal, but in mice, proliferation extends to 2 weeks after birth.

Male reproductive function is largely determined in the early stages of testicular development. The testis differentiates from a genital ridge, from which the structure and cellular organization of the testis originate. Somatic cells first differentiate into Sertoli cells in the early bipotential gonad, which initiates male-specific development and leads germ cells into the spermatogenic lineage. A critical event is the formation of seminiferous cords, which are precursors of seminiferous tubules (Combes et al., 2009). Peritubular myoid cells encircle Sertoli cells and germ cells to form the seminiferous cord (Combes et al., 2009). At the same time, Sertoli cells coordinate the differentiation of other somatic cells (e.g., Leydig cells) that produce the testosterone required to stimulate the proliferation of Sertoli cells. Knockout of the androgen receptor results in a reduction in Sertoli cell numbers by approximately half at birth, and experimental studies in rodents have confirmed the importance of fetal testosterone in determining the Sertoli cell number (Johnston et al., 2004; Scott et al., 2007). Sertoli cells can proliferate in the fetal period, in the neonatal period, and up to 2 weeks of age in rodents (Figure 1) (Sharpe et al., 2003). Sertoli cells are crucial for future reproductive functions because their number determines the capacity for sperm production (Singh and Handelsman, 1996).

In most mammals, including mice and humans, the germline is epigenetically determined, and primordial germ cells are the first cells of the germ lineage. In mice, primordial germ cells are generated from the epiblast and reach the fetal gonads at approximately gestational day (GD) 7.5. They form the first distinctive subpopulation of embryonic cells, which subsequently give rise to spermatogonial stem cells (SSCs) responsible for daily sperm production. Similar to other adult tissue stem cells, SSCs are rare, comprising only 0.03% of the total germ cells in mice (Tegelenbosch and De Rooij, 1993). SSCs proliferate briefly after sex determination in the fetal mouse testis and then enter a prolonged quiescent period from ~GD14.5 until postnatal day 1 or 2 (Vergouwen et al., 1991; Western et al., 2008). In the postnatal period, SSCs are the source of spermatogenesis and are therefore essential for male fertility (Phillips et al., 2010). SSCs can self-renew to maintain the stem cell pool and give rise to a large number of spermatogonia, thereby initiating spermatogenesis (De Rooij, 2017).

## Spermatogenesis

**Figure 2:**
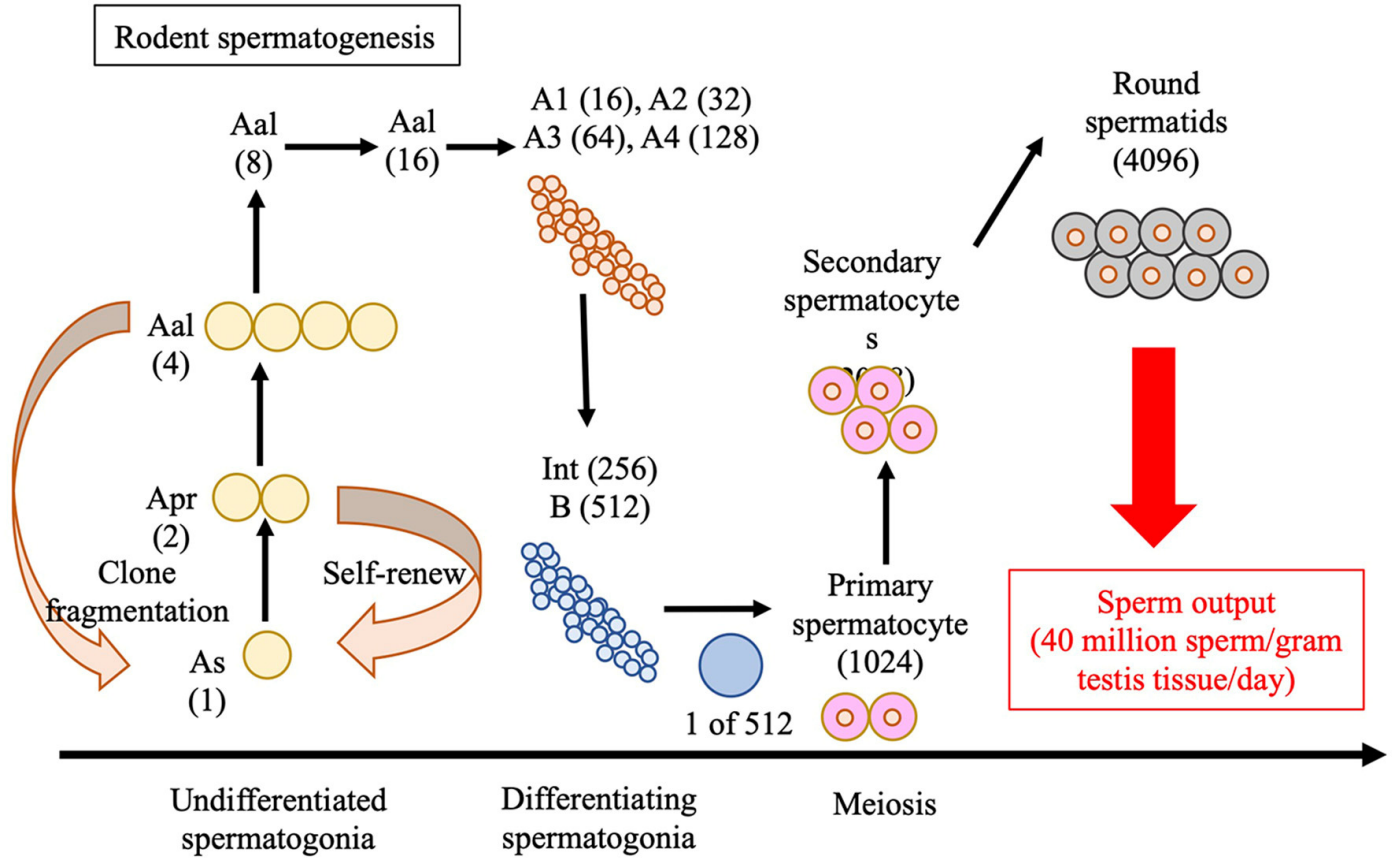
Schematic of spermatogenesis in rodents. “A-single” (As), “A-paired” (Apr), and “A-aligned” (Aal) represent undifferentiated spermatogonia in the 1-, 2-, and 4-cells, respectively. During spermatogenic development, As spermatogonium undergoes one or more mitotic divisions to give rise to larger clones (chains) of interconnected cell sizes through transit-amplifying mitotic divisions. The image features 3–4 transit-amplifying divisions in the pool of undifferentiated As, Apr, and Aal spermatogonia, followed by 6 amplifying divisions in the pool of differentiated spermatogonia (A1–A4, Intermediate, and B-type) that give rise to primary spermatocytes. Two additional meiotic divisions produce the round spermatids that undergo spermiogenesis to produce sperm. Int, Intermediate.

Spermatozoa are produced in the seminiferous tubules of the testes in a process known as spermatogenesis, as illustrated in Figure 2, for rodents. The seminiferous epithelium is lined by tubules, which consist of germ cells and Sertoli cells, and are surrounded by a layer of peritubular myoid cells that provide structural support. Sertoli cells maintain the integrity of the seminiferous cycle and support spermatogenesis by interacting with germ cells (Russell et al., 1990; Nakata et al., 2017). The space between the seminiferous tubules, referred to as the interstitial tissue, contains Leydig cells, blood, and lymphatic vessels, and macrophages (Nakata et al., 2017). Mammalian spermatogenesis is divided into four phases: mitosis of the spermatogonia, meiosis of spermatocytes, elongation of the haploid spermatids (spermiogenesis), and release of the spermatozoa into the lumen of the seminiferous tubules (Oakberg, 1956a,b; Monesi, 1962; Anderson et al., 2008). In mice, the seminiferous cycle is subdivided into 12 stages, known as stages I to XII (Oakberg, 1956a,b).

Endocrine regulation of spermatogenesis occurs through the interplay between gonadotropins and steroids. It is well-known that the hypothalamus controls fertility through the release of gonadotropin-releasing hormone (GnRH), which acts on the pituitary gland to stimulate the secretion of follicle-stimulating hormone (FSH) and luteinizing hormone, which are critical for germ cell development (O'shaughnessy et al., 2009). Luteinizing hormone is responsible for triggering Leydig cells to produce and secrete testosterone, and both FSH and testosterone exert their effects, which are critical for the normal progression of spermatogenesis, directly on Sertoli cells via the FSH receptor (FSHR) and androgen receptor, respectively (Clermont, 1972). In both mice and humans, mutations in the FSHR gene lead to reduced sperm counts, impaired sperm motility, and a low testicular volume, which is attributed to a reduction in Sertoli cell numbers (Tapanainen et al., 1997; Dierich et al., 1998). The effect of testosterone on spermatogenesis can also be mediated by Sertoli cells (Johnston et al., 2001). The progression of late round spermatids to elongating spermatids is sensitive to the loss of Sertoli cell androgen receptor function, but spermatocyte development and progression through meiosis remain unaffected (De Gendt et al., 2004; Holdcraft and Braun, 2004; Denolet et al., 2006). The results of these studies confirm that spermatogenesis requires the action of both FSH and testosterone on Sertoli cells, but the initial entry of germ cells into meiosis seems to be independent of the direct action of either hormone.

In vitamin A-deficient rodents, only undifferentiated spermatogonia and Sertoli cells have been observed within the seminiferous epithelium (Mitranond et al., 1979; Griswold et al., 1989; Van Pelt and De Rooij, 1990a,b; Anderson et al., 2008). Supplying retinoic acid (an active metabolite of vitamin A) to vitamin A-deficient rodents initiated both spermatogonial differentiation and meiosis in male germ cells with the induction of a germ cell-specific retinoic acid response gene, stimulated by retinoic acid gene 8 (Van Pelt and De Rooij, 1990a,b; Anderson et al., 2008), as well as early spermatogenesis, followed by meiosis. We recently demonstrated that chronic vitamin A excess affects spermatogenesis and subsequently impacts sperm parameters in mice (Yokota et al., 2019, 2021). This transition of undifferentiated spermatogonia into differentiated spermatogonia occurs at stages VII–VIII, and meiosis is initiated in preleptotene spermatocytes at the same stages to give rise to leptotene and zygotene spermatocytes at stages IX to XII (Oakberg, 1956a).

The first wave of spermatogenesis does not rely on SSC functions, which indicates that the first complement of spermatogenesis is derived directly from this first group of spermatogonia (Yoshida et al., 2006b). In subsequent waves, SSCs provide a consistent source of progenitor spermatogonia. The undifferentiated spermatogonia, which are diploid, undergo differentiation, and further rounds of mitotic division to give rise to spermatocytes, which then enter meiosis. During the meiotic prophase, recombination occurs, and through two sequential divisions, paternal and maternal chromosomes are distributed randomly into haploid daughter cells, which are termed round spermatids. Finally, these round spermatids develop into elongated spermatids that are released into the seminiferous tubule lumen (Russell et al., 1990).

## Sperm Cell Maturation

Spermatozoa in the seminiferous tubules do not possess motility and the potential to fertilize an oocyte. In mammals, sperm cells mature, and become fertile as they traverse a specialized duct called the epididymis (Bedford et al., 1973; Cooper, 2007). In mice, the epididymis is composed of three main segments: the caput (proximal), corpus (middle), and cauda (distal) epididymis (Nakata and Iseki, 2019). The caput epididymis is highly regulated by androgens, with the highest concentrations of the androgen-metabolizing enzyme 5-alpha-reductase, which converts testosterone to dihydrotestosterone (Viger and Robaire, 1991). Several studies have shown that each segment of the epididymis has a high degree of transcriptional differentiation, which is likely linked to the sequential role of each segment in sperm maturation (Viger and Robaire, 1991; Ezer and Robaire, 2003; Johnston et al., 2007; Turner et al., 2007). These segments are regulated by endocrine, lumicrine, and paracrine factors. Efferent ductules and the caput epididymis are dependent on the estrogen signaling required for ion transport, water reabsorption, and regulation of sperm concentrations (Cooke et al., 2017). The caput epididymis secretes numerous proteins, such as ovochymase 2 (required for processing of the sperm surface protein A disintegrin) and metallopeptidase 3 (which gives the sperm the ability to fertilize oocytes) (Kiyozumi et al., 2020). The corpus epididymidis absorbs substances from the lumen of the epididymis, including the sperm droplets that are shed during transit through the corpus (De Grava Kempinas and Klinefelter, 2014). Finally, the sperm cells are stored in a quiescent state in the cauda epididymis, where they remain until ejaculation. Transit of mouse sperm through the epididymis requires ~10 days (Conine et al., 2018). In the next section, we describe the effects of the developmental toxicity of NMs on reproductive functions via changes in small non-coding RNA expression.

## Effects of Exposure to Nanomaterials on Male Reproductive Systems

Engineered NMs have gained importance with respect to technological applications over the past few years. The rapid advancement of nanotechnology has led to concerns about the potential risks that NMs pose to human health by increasing the overall particle burden in air for workers and consumers, and thus, there is a need to investigate the health effects of exposure to NMs containing particles and fibers. According to the European Commission, NMs are natural, accidental, or manufactured materials with one or more dimensions at the nanoscale, where nanoscale is defined as a size ~100 nm (Boverhof et al., 2015).

The introduction of NMs with novel physicochemical characteristics into the environment may result in unexpected toxicity that is not caused by the bulk material. NMs have been shown to easily cross the epithelial cells and the walls of small capillaries. Thus, following exposure, NMs can circulate freely throughout the body and pass through blood–organ barriers. For example, unlike bulk materials, NMs can penetrate the blood–testis barrier and reach the seminiferous epithelium (Kim et al., 2006), potentially affecting reproductive health (Hougaard et al., 2015; Ema et al., 2017). Moreover, exposure to NMs in adulthood has also been shown to adversely affect male reproductive function (Yoshida et al., 1999, 2009; Izawa et al., 2007; Li et al., 2012; Jia et al., 2014; Skovmand et al., 2018; Lauvås et al., 2019; Miura et al., 2019). In mice that were orally administered silver-NMs (22, 42, and 71 nm in size) or large-sized materials (323 nm) at a dose of 1 mg/kg body weight for 2 weeks, the silver-NMs caused an accumulation of silver within the testes, whereas the large-sized materials did not (Park et al., 2010). A study demonstrated a significant accumulation of NMs in the testes of mice that had been intraperitoneally administered 50-nm silica-coated magnetic NMs (10, 25, 50, or 100 mg/kg) for 4 weeks (Kim et al., 2006). Moreover, in mice that had inhaled filtered air (control), a low dose (44.89 × 10^5^ ± 2.37 × 10^4^ particles/cm^3^), or high dose (9.34 × 10^5^ ± 5.11 × 10^4^ particles/cm^3^) of 50-nm fluorescent magnetic NMs for 4 weeks (4 h/day, 5 days/week), NMs were frequently detected in the testes, suggesting that magnetic NMs after inhalation could also pass through the blood–testis barrier (Kwon et al., 2008). Researchers administered three sizes (14, 56, and 95 nm) of carbon black-NMs intratracheally to ICR male mice (0.1 mg/mouse, 10 times a week) to investigate their adverse effects on male reproduction (Yoshida et al., 2009). They found that the mice exposed to the 14-nm NMs had less partial vacuolation of the seminiferous tubules than those exposed to the 56-nm NMs, despite the number of particles administered being approximately the same, suggesting that the toxicity to male reproduction was because of the particle size rather than the particle number (Yoshida et al., 2009). It is yet unknown whether exposure to NMs in adults can directly or indirectly affect male reproduction. Skovmand et al. (2018) used a mouse model of pulmonary inflammation to indicate the presence of carbonaceous NMs in bronchoalveolar lavage fluid. However, after the intratracheal instillation of three different carbonaceous NMs to male mice (0.1 mg/mouse) once a week for seven consecutive weeks, no significant changes in epididymal sperm parameters, daily sperm production, or plasma testosterone levels were noticed in any of the three exposure groups (Skovmand et al., 2018). NM accumulation was not observed in the testes of mice that were injected intravenously with titanium dioxide-NMs (TiO_2_-NMs) at doses of 0.1, 1, 2, or 10 mg/kg body weight, although sperm parameters were affected by TiO_2_-NM exposure (Miura et al., 2017). Moreover, in a follow-up study by the same authors, a single intratracheal administration of TiO_2_-NMs (at a dose of 100 or 500 μg/mouse) showed remarkable effects on sperm motility in mice (Miura et al., 2019). As mentioned in the explanation of germ cell development below, because 35 days are required for spermatogenesis in the testes, followed by 10 days for sperm maturation in the epididymis, short-term effects may actually have direct effects on the epididymis.

Several studies have been carried out to understand the potential mechanisms underlying the toxic effects of NMs on male reproduction. For example, NM suspensions have been incubated with Sertoli cell line (TM-4), spermatocyte cell line (GC2-spd) (Liu et al., 2016), primary cultured Sertoli cells isolated from mice (Hong et al., 2016), and mature spermatozoa (Yoisungnern et al., 2015; Özgür et al., 2018; Préaubert et al., 2018; Cotena et al., 2020) to evaluate whether they have direct cytotoxic effects. Silver-NMs were found to interfere with the proliferation of mouse SSCs in a dose-dependent and particle size-dependent manner, and small-sized NMs (10–25 nm in diameter) were more likely to promote apoptosis or production of reactive oxygen species (ROS) in these cells than the large-sized (80–130 nm in diameter) NMs (Braydich-Stolle et al., 2005). However, because these *in vitro* or *ex vivo* studies do not seem to cover the mechanism underlying the toxicity of NMs toward male reproductive functions, predictions of nanotoxicity *in vivo* cannot be made.

## Effects of Maternal Exposure to Nanomaterials on the Reproductive System in Male Offspring

It is well-known that the fetus may be more sensitive to exposure to chemical substances than adults (Barouki et al., 2012; Heindel et al., 2015). This concept was covered as the “Developmental Origins of Health and Disease (DOHaD) hypothesis” on the basis of accumulating evidence from epidemiological and experimental studies demonstrating that the early life environment, especially *in utero* and assisted reproductive technology, can easily decide or influence health and disease risks later in life (Carpinello et al., 2018).

Thus, there is a need to investigate the effects of engineered NMs on reproductive and developmental health. To the best of our knowledge, our previous work was the first to show that maternal inhalation of diesel exhaust (DE: 1.0 mg/m^3^, from gestational days 2 to 16) increases testosterone levels and affects the expression of genes involved in testosterone synthesis (Yoshida et al., 2006a). A subsequent study demonstrated that DE inhalation during the fetal period affects spermatogenesis (Ono et al., 2007). We showed that these effects were caused by nano-sized PMs (diameter <100 nm), including DE (Ono et al., 2008). These results indicated that maternal inhalation of nano-sized PMs could directly and/or indirectly act on germ cells in the fetal period. In view of these findings, we evaluated the developmental toxicity of NMs and showed for the first time that 35 nm TiO_2_-NMs that had been repeatedly administered to pregnant mice (at a dose of 0.1 mg) on gestational days 3, 7, 10, and 14 were able to pass from mother to pups, and we confirmed their distribution in the brain and testis (Takeda et al., 2009). In this exposure model, we demonstrated that maternal exposure to TiO_2_-NMs reduced daily sperm production and observed pathological findings in the testes of the male offspring (Kubo-Irie et al., 2014).

Other studies have also reported the effects of prenatal exposure to NMs on reproductive health (Ono et al., 2008; Yoshida et al., 2010; Skovmand et al., 2019). However, evidence regarding the developmental toxicity of engineered NMs is limited and remains insufficient for risk assessment in pregnant women and their children (Hougaard et al., 2015). Furthermore, the route of NMs from the port of entry to the fetus is not clearly known, although partial accumulation of NMs in the fetus has been observed (Takeda et al., 2009; Valentino et al., 2016; Campagnolo et al., 2017), and it remains to be investigated whether the effects on fetuses are due to the direct actions of NMs and how the developmental toxicity of NMs causes defects in male reproductive function later in life. Chronic inflammation could also be a potential mechanism of indirect toxicity, since it is a candidate mechanism of several disorders in adults (Khansari et al., 2009). Maternal inflammation caused by ROS could interfere as well with fetal development and affect the offspring later in life (Romero-Haro and Alonso-Alvarez, 2020). In fact, in a study by Sager and Castranova (2009) conducted on rats, pulmonary exposure to small-sized NMs induced a systemic inflammatory event that was generally more pronounced than that elicited by large-sized NMs (Sager and Castranova, 2009). In another study, pregnant mice from gestational days 0.5 to 14.5 were exposed to 18–20 nm silver-NMs (at mass concentration 640 μg/m^3^) by nose-only inhalation for 4 h/day (Campagnolo et al., 2017). This study simulated the lung burden to calculate the accumulation of silver-NMs in the lungs, whereupon an accumulated dose of 270 mg/kg lung weight was estimated. Many NMs are characterized by their high surface reactivity, which demonstrates a high inherent potential for inducing inflammation and generating ROS at the site of deposition (i.e., lung deposition) (Hougaard et al., 2010; Madl et al., 2014). It is possible that circulating inflammatory cytokines and ROS may attack the placenta and embryo/fetus. However, Campagnolo et al. (2017) found that silver-NMs inhaled by pregnant mice during the fetal period were able to reach the placenta and increase the gene expression of pro-inflammatory cytokines (TNFα and IL-1β), subsequently decreasing fetal growth (Campagnolo et al., 2017). Therefore, there is no conclusive evidence on whether the effects of the developmental reproductive toxicity of NMs on the next generation are due to direct and/or indirect mechanisms. Finally, given the previous finding that the surfaces of the NMs are immediately coated with proteins (protein corona) (Ban et al., 2020), it is important to evaluate the composition of biomolecules interacting with the surface of NMs in different organs in order to understand the distribution of NMs and to investigate their potential toxicity and the mechanisms causing them. In the next section, we discuss the potential molecular toxicity mechanisms of NM exposure.

## MicroRNAs as a Potential Early Biomarker of Developmental Toxicity in the Testes Mediated by Prenatal NM Exposure?

MicroRNAs (miRNAs), a class of short (20–25 nucleotides) non-coding RNAs, are well-documented as critical post-transcriptional regulators of gene expression that act by causing the degradation of a target mRNA and hence the repression of translation (Papaioannou and Nef, 2010). The mRNA target sequence can possess multiple binding sites for different miRNAs, and a single miRNA can have hundreds of target mRNAs. Thus, miRNAs have been shown to regulate a broad range of different targets and are involved in various toxicological processes and diseases (Sood et al., 2006). The transient inhibition of miR-21 in SSC-enriched germ cell cultures increased the number of germ cells undergoing apoptosis and reduced stem cell potency, suggesting that miR-21 is important for maintaining SSCs (Niu et al., 2011). MiR-20 and miR-106a, preferentially expressed in SSCs, have been shown to be essential for the renewal of SSCs (He et al., 2013). The levels of miR-146 were highly elevated in undifferentiated spermatogonia and were downregulated by retinoic acid to induce the differentiation of spermatogonia, suggesting that miR-146 suppresses retinoid acid-induced spermatogonial differentiation (Huszar and Payne, 2013). The X chromosome-clustered miR-221 and miR-222, which are highly expressed in undifferentiated cells, were also downregulated by retinoic acid treatment (Yang et al., 2013), and the loss of function of miR-221 and miR-222 in mice induces differentiation and the loss of stem cell capacity (Yang et al., 2013).

In mammalian spermatogenesis, extensive transcriptional activity occurs in pachytene spermatocytes and round spermatids (Soumillon et al., 2013), indicating that miRNAs also play a critical role in meiotic and post-meiotic spermatogenic cells. MiR-34c is important for the later stages of spermatogenesis because its expression is highly up-regulated in spermatocytes and round spermatids (Bouhallier et al., 2010; Romero et al., 2011; Liang et al., 2012). Other miRNAs involved in the regulation of meiotic and post-meiotic gene expression include the miR-449 cluster of miRNAs, which are significantly upregulated at meiotic initiation (Bao et al., 2012).

The successful compaction of sperm chromatin by the replacement of testis-specific histone variants and transition proteins with protamines is required for spermiogenesis (Bao et al., 2012). The expression of these proteins is also regulated by miRNA-mediated mechanisms. MiR-469 has been shown to target transition protein-2 and protamine-2 mRNAs and thus repress their translation in pachytene spermatocytes and round spermatids through mRNA degradation (Dai et al., 2011). MiR-122a, expressed in late-stage male germ cells, also induces transition protein-2 mRNA degradation, which causes a reduction in the protein levels of transition protein-2 expression (Yu et al., 2005).

miRNAs show tissue-specific and developmentally regulated expression patterns, and dysregulation of a given miRNA may cause male infertility. However, although changes in the expression ratio of the protamine-1 and−2 transcripts in infertile patients have been reported (Lambard et al., 2004; Steger et al., 2008), there are currently no data available regarding pairs of miRNAs in sperm with a stable or disrupted expression ratio in relation to the fertility status. Further investigation is needed to search for sensitive biomarkers of miRNAs involved in fertility and/or testicular toxicity. Moreover, there are no data on the effects of prenatal exposure to NMs on the expression levels of miRNAs in the testes of offspring. However, a recent study has reported that long-term exposure to concentrated ambient PM_2.5_ affects sperm miR-6909-5p expression in male mice (Chen et al., 2021).

## Transfer of Sperm MicroRNAs to Oocytes to Regulate Development

The importance of miRNAs during spermatogenesis has been previously described. Recently, it has also been reported that sperm borne miRNA expression can be transferred to the zygote and that paternal miRNA expression regulates zygotic gene activation, which could affect embryo development (Liu et al., 2012). Moreover, mice exposed to traumatic stress in early life altered the expression of miRNAs in sperm cells. Injection of sperm RNAs from these traumatized males into fertilized wild-type oocytes resulted in offspring with neurobehavioral alterations similar to those of the male parent (Gapp et al., 2014). After the sperm enters the oocyte, it delivers a variety of RNA species (Liu et al., 2012; Yuan et al., 2016; Conine et al., 2018). In one study, zygotes were produced from miRNA-depleted sperm in Drosha conditional knockout mice using intracytoplasmic sperm injection. These zygotes had reduced developmental potential, which was recovered through the injection of miRNAs from wild-type sperm (Yuan et al., 2016). Thus, several miRNAs in sperm seem to affect the activation of minor/major zygotic genes during early embryonic development. However, the effects of individual sperm miRNAs delivered to zygotes are still unknown. Recently, miR-34 has been found to initiate the first cleavage division in mice (Liu et al., 2012), which is a distinctive example of the impact of a single paternal miRNA on embryonic development.

**Figure 3:**
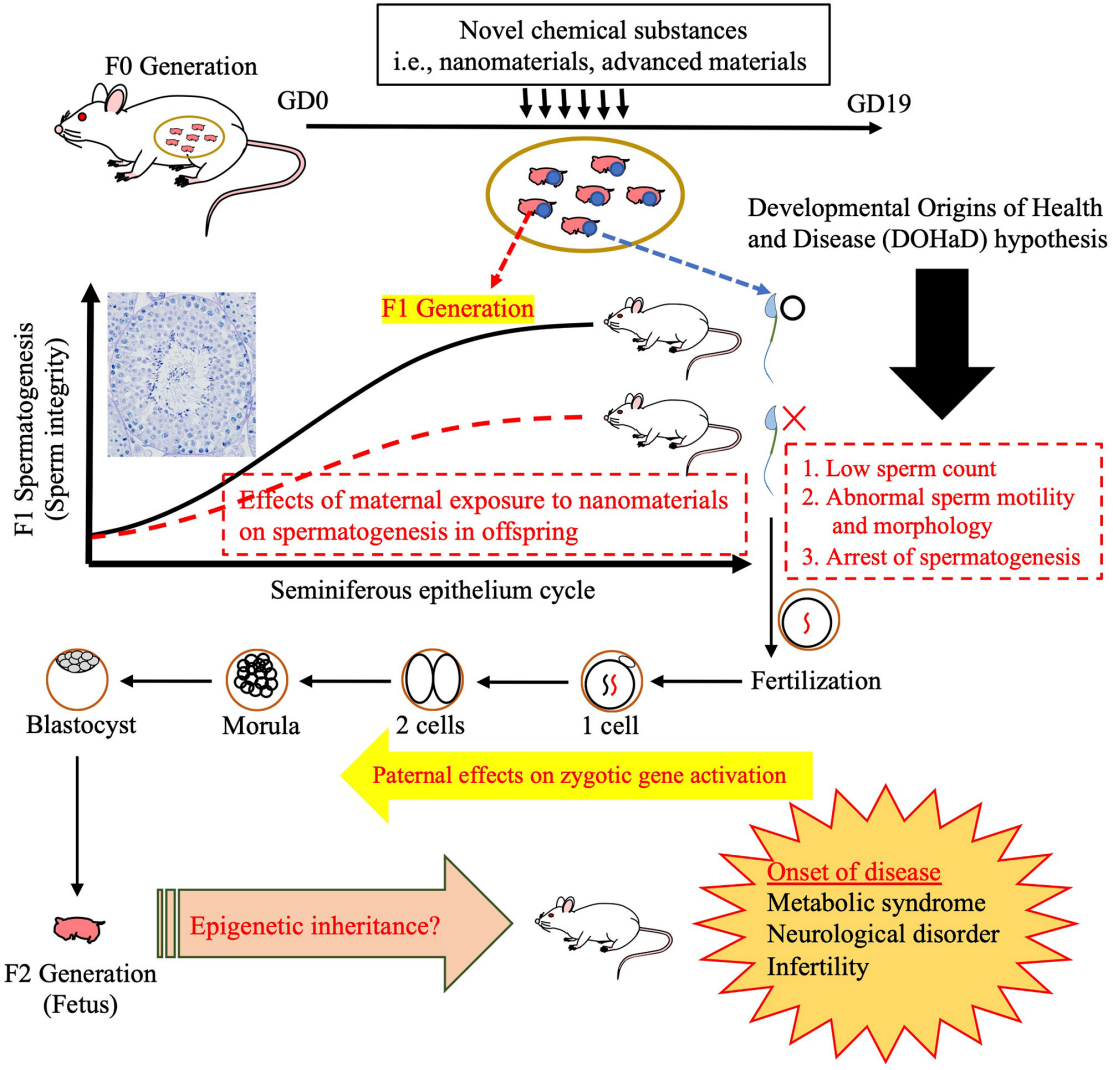
Illustration of intergenerational (F1) and transgenerational (F2) effects. The testis morphology shown demonstrates the cooperation of somatic cells and spermatogenic cells during spermatogenesis. Schematic of susceptibility windows for testicular toxicity in the F1 generation caused by prenatal exposure to nanomaterials (NMs), followed by the induction of potential toxicity mechanisms via the F1 germline to trigger transgenerational inheritance to the F2 generation, which is not subject to direct NM exposure. After mating with control females, phenotypic alterations are often observed in offspring in these paradigms. Illustrated here are a number of probable disease onset mechanisms underlying such paternal effects, including alterations in the sperm epigenome.

**Figure 4:**
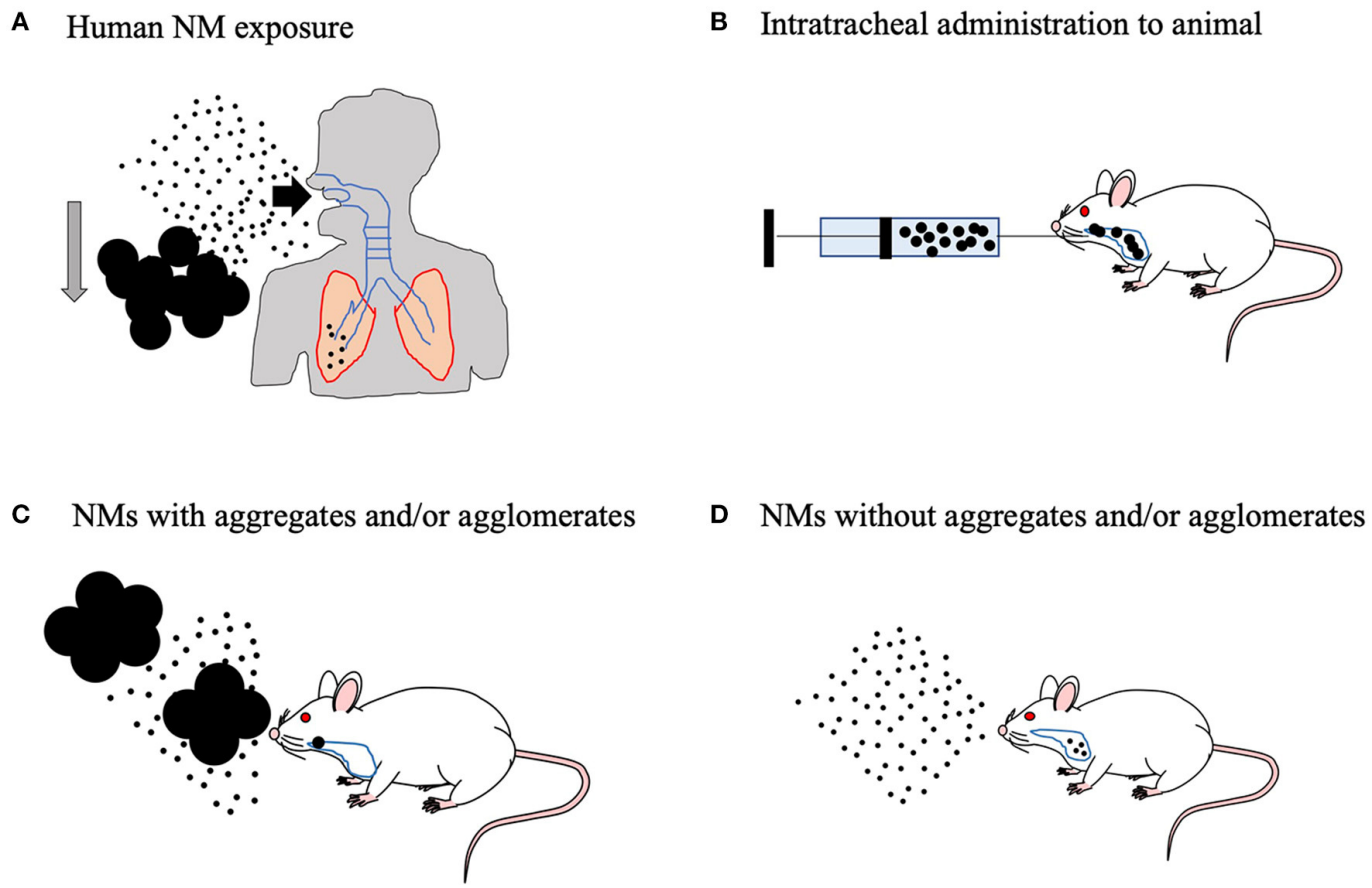
Animal inhalation experiment to predict the effects of human-relevant exposure. **(A)** The aggregates and/or agglomerates will sediment quickly in ambient air and will be filtered out effectively by the human upper respiratory tract. Humans can be easily exposed to well-dispersed nanomaterials (NMs) in the environment. In fact, human exposure to well-dispersed NMs can result in alveolar lesions. **(B)** Therefore, suspension of the NMs in biological fluid with aggregates and/or agglomerates does not show the full extent of the effects of NMs on experimental animals. **(C)** On the other hand, in practical inhalation studies on experimental animals, even though the air in the animal chamber is rigorously agitated to ensure homogeneity of the aerosols, NMs with aggregates and/or agglomerates can enter the respiratory tract of the animal, which makes nanotoxicity evaluation difficult. **(D)** To address this problem, we developed aerosolized, well-dispersed NMs without aggregates, and/or agglomerates.

One group have reported that maternal exposure to NMs affects daily sperm production in both the F1 and F2 generations (Kyjovska et al., 2013). This finding point to the possibility that maternal NM exposure affects spermatogenesis in the F1 generation, followed by sperm-derived (paternal) effects on development in the F2 generation (Figure 3). In fact, we discovered changes in miRNA expression in F1 sperm following the maternal inhalation of NMs, which were administered using the high dispersion method (Figure 4). Furthermore, we observed abnormal neurobehavior and decreased sperm production in the F2 generations (data not shown). In a recent study, Chen et al. (2021) found that sperm miR-6909-5p expression was affected in male mice that had been exposed to concentrated ambient PM_2.5_ for 12 weeks, and the injection of miR-6909 into the zygote could mimic the effects of paternal PM_2.5_ exposure on energy homeostasis in the offspring (Chen et al., 2021). However, there was a few evidences about transfer of sperm-borne RNA to the next generation caused by maternal NM exposure. Further investigation is needed to identify the cause of these transgenerational effects before the use of sperm RNA as a biomarker of the developmental toxicity of NMs.

## Perspective: Future Evaluation of Nanotoxicity Using a Human Relevant Exposure Method

NMs suspended in the environment are easily incorporated into the body, primarily via the airway (Figure 4A). For the assessment of nanotoxicity, an experiment using an inhalation method is therefore needed. Intratracheal methods are often used as surrogates for inhalation exposure. NMs were solubilized in physiological buffer to perform intratracheal instillation. However, it could cause excessive aggregation and agglomeration to properly evaluate nanotoxicity in rodents, because the high salt concentration in the buffer leads to improper NM dispersion (Fu et al., 2014; Abdelgied et al., 2019). Although NMs (specifically TiO_2_ nanoparticles) were stabilized and well-dispersed in alkaline or acidic buffers, they were substantially different from biological fluids in terms of pH (Kobayashi et al., 2009; Guiot and Spalla, 2013). Therefore, these animal experiments could not be used to evaluate the safety of human-relevant exposure (Figure 4B). Additionally, the general inhalation method may also cause aggregates or agglomerates of NMs (Figure 4C) (Yoshida et al., 1999). Our laboratory is now attempting to develop a novel inhalation chamber to expose highly dispersed NMs to rodents in order to replicate relevant human exposure scenarios (Figure 4D) (Taquahashi et al., 2013; Otsuka et al., 2018). The human male reproductive system is vulnerable to many exogenous materials (Howards, 1995). Therefore, further investigations are needed to assess the risks related to maternal exposure to NMs through inhalation and to determine whether transgenerational effects occur via sperm of the F1 generation exposed during the fetal period.

## Author Contributions

SY drafted the manuscript. SY, KT, and SO critically revised the article for important intellectual content. All authors contributed to the article and approved the submitted version.

## Conflict of Interest

The authors declare that the research was conducted in the absence of any commercial or financial relationships that could be construed as a potential conflict of interest.
